# Correction to “Surgical Resection for Rare Cases of Lung Cancer in Teenagers”

**DOI:** 10.1002/rcr2.70340

**Published:** 2025-09-09

R. Sumiya, T. Matsunaga, Y. Watanabe, et al., “Surgical Resection for Rare Cases of Lung Cancer in Teenagers,” *Respirology Case Reports* 13, no. 8 (2025): e70281, https://doi.org/10.1002/rcr2.70281.

Figure 2 is incorrect as the pathological findings were not representative images. Below is the correct Figure 2.

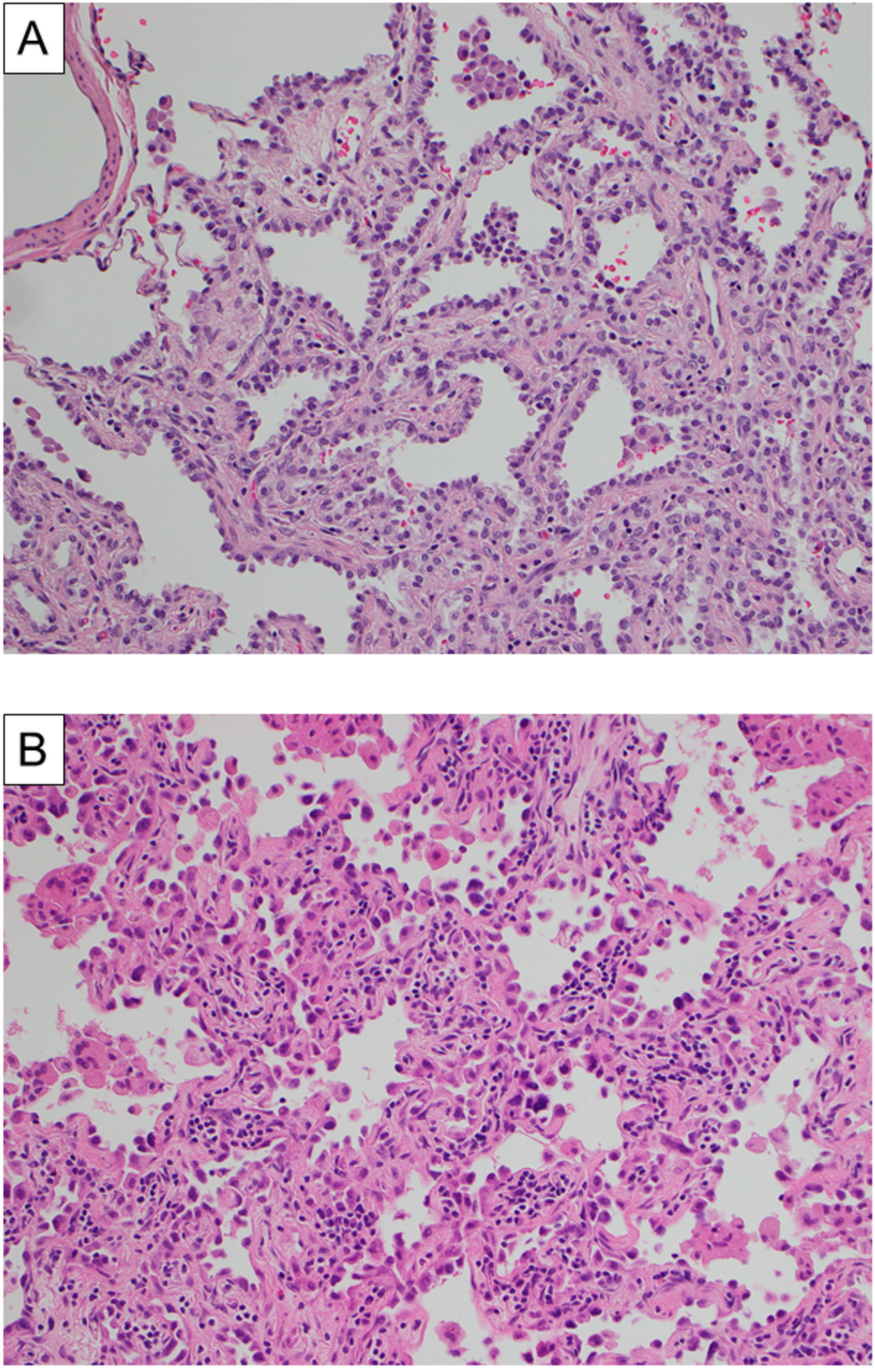

We apologize for this error.

